# Supplementary material for: Comparing the effect of childbirth preparation courses delivered both in-person and via social media on pregnancy experience, fear of childbirth, birth preference and mode of birth in pregnant Iranian women: A quasi-experimental study
Source: PLoS One. 2022 Aug 5;17(8):e0272613. doi: 10.1371/journal.pone.0272613 (PMC9355199; doi:10.1371/journal.pone.0272613)
Supplement: S1 Protocol — (PDF) [file pone.0272613.s002.pdf]

Back (https://en.irct.ir/user/profile)

Effect of Childbirth Education based on Face to Face and the Social Network on Pregnancy and Delivery Experience and Type of Delivery on Pregnant Women

✔ Approved

Trial Id

30890

IRCT Id

IRCT20180427039436N2

Registration date

2018-06-15, 1397/03/25

Registration date

2018-06-15, 1397/03/25

Membership number

39436

If you have a feedback for the referee or an explaination regarding referee messages/entered data, please leave it here.

Save message

General information

Reason for update

فا empty

En empty

Scientific title

فا مقایسه تأثیر آموزش آمادگی برای زایمان به دو شیوه حضوری و مبتنی بر شبکه اجتماعی بر تجربه بارداری، زایمان و نوع زایمان در زنان باردار

En Effect of Childbirth Education based on Face to Face and the Social Network on Pregnancy and Delivery Experience and Type of Delivery on Pregnant Women

Public title

فا بررسی تاثیر آموزش مجازی آمادگی برای زایمان در زنان باردار

En Effect of virtual Childbirth Education on pregnant women

Acronym

empty

Study design

Interventional

Phase

N/A

Minimum age

18 year

Maximum age

35 year

Gender

Female

Inclusion criteria

فا داشتن ملیت ایرانی
نخست زا بودن
زنان 35-18 ساله
سن حاملگی ۲۰ هفته
توانایی خواندن و نوشتن
داشتن تلفن همراه یا کامپیوتر با قابلیت نصب برنامه تلگرام تا پایان مطالعه
دسترسی به اینترنت فعال
عدم وجود هر گونه بیماری یا شرایط خاصی که فرد را در گروه بارداری پر خطر قرار دهد
عدم سابقه ناباروری و بیماری های روانی

En Iranian nationality
nullipara
18-35 years old
20 week of gestational age
Literate
Having a mobile phone or computer with the ability to install a telegram program until the end of the study

Active Internet access
Absence of high risk pregnancy
Lack of history of infertility and psychological disorder

Exclusion criteria

فا عدم حضور در بیش از ۲ جلسه از کلاس های آمادگی زایمان
ترک گروه به هر دلیلی (انصراف نمونه ها از ادامه شرکت در مطالعه)
بازخورد ندادن نسبت به پیام های ارسال شده توسط پژوهشگر به مدت حداقل یک هفته طی مطالعه
بروز هر گونه علائم خطر بارداری در طی مطالعه که فرد را به گروه پرخطر وارد سازد
زایمان زودرس

En Being absent more than two sessions of childbirth classes
Leave the group
Not responding to messages sent by the researcher for at least one week during the study
Indication of high risk pregnancy during study
Preterm labor

Randomization (investigator's opinion)
Not randomized

Randomization description

فا *empty*

En *empty*

Blinding (investigator's opinion)
Not blinded

Blinding description

فا *empty*

En *empty*

Placebo
Not used

Assignment
Parallel

Purpose
Education/Guidance

Other design features

فا *empty*

En *empty*

Sample size
Target sample size
Target sample size: **165**
More than 1 sample in each individual: **No**
Number of samples in each individual: ***empty***
Description of samples in each individual - Persian: فا ***empty***
Description of samples in each individual - English: En ***empty***

Actual sample size reached
Actual sample size reached: ***empty***
More than 1 sample in each individual: **No**
Actual sample size in each individual: ***empty***
Description of samples in each individual - Persian: فا ***empty***
Description of samples in each individual - English: En ***empty***

Expected recruitment start date
2018-07-06, 1397/04/15

Expected recruitment end date
2019-08-21, 1398/05/30

Actual recruitment start date
empty

Actual recruitment end date
empty

Trial completion date
empty

Secondary Ids

empty

Ethics committees

Ethics committee

Name of ethics committee
Ethics committee of Iran University of Medical Sciences
کمیته اخلاق دانشگاه علوم پزشکی ایران

Street address
Rashid Yasemi
رشید یاسمی

City
Tehran
تهران

Province
Tehran
Postal code
1996713883

Approval date
2018-02-25, 1396/12/06

Ethics committee reference number
IR.IUMS.REC 1396.9511373011

Health conditions studied

Description of health condition studied

فا آموزش مجازی آمادگی برای زایمان

En Virtual education of childbirth preparation

ICD-10 code
empty

ICD-10 code description
empty

Primary outcomes

Description

فا تجربه بارداری

En pregnancy experience

Timepoint

فا پیش از مداخله در هفته 16-20 بارداری و در هفته 36-38 بارداری

En Before intervention at 16-20 weeks gestational and 36-38 weeks of gestational age

Method of measurement

فا پرسشنامه کوتاه شده تجربه بارداری

En Brief version of pregnancy experience scale

Description

فا انتظار/ تجربه زایمانی

En delivery expectancy/experience questionnaire

Timepoint

فا پیش از مداخله در هفته 16-20 بارداری و در هفته 36-38 بارداری

En Before intervention at 16-20 weeks gestational and 36-38 weeks of gestational age

Method of measurement

فا پرسشنامه انتظار/ تجربه زایمانی

En Wijma delivery expectancy/experience questionnaire

Description

فا نوع زایمان

En Type of delivery

Timepoint

فا پس از زایمان

En After delivery

Method of measurement

فا پرسش مبتنی بر شبکه اجتماعی

En Question based on social networking

Secondary outcomes

empty

Intervention groups

Description

فا آموزش مبتنی بر شبکه اجتماعی (تلگرام): محتوای آموزشی منطبق بر دستورالعمل استاندارد کشوری بوده و ارسال پیام ها بر اساس اصول چندرسانه ای مایر و به صورت متن، تصویر، پادکست و ویدئوکست در فرمت MPEG-4، خواهد بود. حداکثر مدت زمان فیلم های آموزشی، 10-15 دقیقه خواهد بود.

En Education based on social networking (on Telegram): The educational content is according to country standard instructions. In addition, sending messages based on Mayer's multimedia in form of text, image, podcast, video cast, in the MPEG-4 format. The maximum of films time will be 10-15 minute.

Category
other

Description

فا کلاس های آمادگی زایمان حضوری: این کلاس ها در 8 جلسه به صورت حضوری در بیمارستان برگزار خواهد شد.

En Face to face childbirth preparation classes: These classes will be held at the hospital in 8 sessions.

Category

other

Description

فا گروه کنترل: عدم شرکت در هر گونه دوره آموزشی

En Control group: Not attending any educational course

Category

other

Recruitment centers

Recruitment center

Name of recruitment center

Milad hospital

بیمارستان میلاد

Full name of responsible person

Robab Mousavi

رباب موسوی

Street address

Shahid Hemmat highway, District 2

منطقه دو، بزرگراه شهید همت

City

Tehran

تهران

Province

Tehran

Postal code

881/14665

Phone

+98 21 84090

Fax

+98 21 8806 2005

Email

info@miladhospital.com

Web page address

Sponsors / Funding sources

Sponsor

Name of organization / entity
Iran University of Medical Sciences
دانشگاه علوم پزشکی ایران

Full name of responsible person
Kazem Malakouti
کاظم ملکوتی

Street address
Shahid Hemmat highway, District 2
منطقه دو، بزرگراه همت

City
Tehran
تهران

Province
Tehran

Postal code
1449614535

Phone
+98 21 6650 9024

Fax
+98 21 8805 2248

Email
malakoutik@yahoo.com

Grant name

فا *empty*

En *empty*

Grant code / Reference number
empty

Title of funding source

فا دانشگاه علوم پزشکی ایران

En Iran University of Medical Sciences

Proportion provided by this source
100%

Public or private sector
public

Domestic or foreign origin
domestic

Person responsible for general inquiries

Name of organization / entity
Iran University of Medical Sciences

دانشگاه علوم پزشکی ایران

Full name of responsible person
Robab Mousavi

رباب موسوی

Position
Student

دانشجو

Latest degree
Master

Other areas of specialty/work
Midwifery

مامایی

Street address
Rashid Yasemi

رشید یاسمی

City
Tehran

تهران

Province
Tehran

Postal code
1996713883

Phone
+98 21 4365 1000

Mobile
+98 910 924 5921

Email

rrr.mousavi@gmail.com

Person responsible for scientific inquiries

Name of organization / entity
Iran University of Medical Sciences
دانشگاه علوم پزشکی ایران

Full name of responsible person
Robab Mousavi
رباب موسوی

Position
Student
دانشجو

Latest degree
Master

Other areas of specialty/work
Midwifery
مامایی

Street address
Rashid Yasemi
رشید یاسمی

City
Tehran
تهران

Province
Tehran

Postal code
1996713883

Phone
+98 21 4365 1000

Mobile
+98 910 924 5921

Email
rrr.mousavi@gmail.com

Person responsible for updating data

Name of organization / entity
Iran University of Medical Sciences
دانشگاه علوم پزشکی ایران

Full name of responsible person
Leila Amiri Farahani
لیلا امیری فراهانی

Position
Assistant professor
استادیار

Latest degree
Ph.D.

Other areas of specialty/work
Reproductive Health
سلامت باروری

Street address
Rashid Yasemi
رشید یاسمی

City
Tehran
تهران

Province
Tehran

Postal code
1996713883

Phone
+98 21 4365 1139

Mobile
+98 912 450 5862

Email
l.amirifarahani@gmail.com

Protocol summary

Study aim

فا مقایسه تأثیر آموزش آمادگی برای زایمان به دو شیوه حضوری و مبتنی بر شبکه اجتماعی بر تجربه بارداری، زایمان و نوع زایمان با گروه کنترل در زنان باردار

En Comparing the Effect of Childbirth Education based on Face to Face and the Social Network on Pregnancy and Delivery Experience and Type of Delivery on Pregnant Women

Participants/Inclusion and exclusion criteria

فا زنان نخست زای واجد شرایط دریافت کننده مراقبت های معمول بارداری

En Nulliparous women are eligible to receive common perinatal cares

Intervention groups

فا در این مطالعه نمونه ها به طور مساوی، در سه گروه مداخله یک (آموزش مبتنی بر شبکه اجتماعی (تلگرام))، مداخله دو (کلاس های آمادگی زایمان حضوری) و کنترل (عدم شرکت در هر گونه دوره آموزشی) قرار خواهند گرفت.

En In this study, participants will assign to one of three groups: intervention group 1 (education based on social networking (on Telegram)), intervention group 2 (face to face childbirth preparation classes) and control group (Not attending any educational course.

Design

فا مطالعه نیمه تجربی دارای گروه کنترل، با سه گروه های موازی، حجم نمونه 165 نفر

En Semi experimental study with control group, with three parallel groups design of 165 patients

Settings and conduct

فا این مطالعه در بخش پری ناتال بیمارستان میلاد انجام خواهد شد. محتوای آموزشی مطالب به اشتراک گذاشته شده منطبق با دستورالعمل استاندارد کشوری بوده و ارسال پیام ها بر اساس اصول چندرسانه ای مایر و به صورت متن، تصویر، پادکست و ویدکست در فرمت MPEG-4، خواهد بود. حداکثر مدت زمان فیلم های آموزشی، ۱۵-۱۰ دقیقه بوده و جهت جلوگیری از ارسال یکباره مطالب آموزشی، محتوای هر جلسه در بخش های تقسیم بندی شده، به صورت روزانه ارسال خواهد شد. جهت اطمینان از آموزش صحیح تکنیک های تنفسی و تن آرامی، دو جلسه برای تمرین حضوری تشکیل خواهد شد.

En This study will be conducted in the perinatal clinic of the Milad Hospital. The educational content is according to country standard instructions. In addition, sending messages based on Mayer's multimedia in form of text, image, podcast and video cast, in the MPEG-4 format. The maximum of films time was 10-15 minute. To prevent from being uploaded, as well as reduction in educational quality, the content of each session in divided sections in that session and specific time will be sent every day. To make sure of correct methods education of respiratory and relaxation techniques, two sessions to practice will be hell in the hospital.

Main outcome variables

فا تجربه بارداری؛ انتظار/تجربه زایمانی؛ تمایل به نوع زایمان؛ نوع زایمان

En Pregnancy experience; Delivery expectancy/experience; Desire to type of delivery; Type of delivery

Sharing plan

Deidentified Individual Participant Data Set (IPD)

Undecided - It is not yet known if there will be a plan to make this available

Justification/reason for indecision/not sharing IPD

فا در اینده تصمیم گیری می شود.

En it is will be known in future.

Study Protocol

No - There is not a plan to make this available
Statistical Analysis Plan
No - There is not a plan to make this available
Informed Consent Form
No - There is not a plan to make this available
Clinical Study Report
No - There is not a plan to make this available
Analytic Code
No - There is not a plan to make this available
Data Dictionary
No - There is not a plan to make this available
Title and more details about the data/document

empty فا

En *empty*
When the data will become available and for how long

empty فا

En *empty*
To whom data/document is available

empty فا

En *empty*
Under which criteria data/document could be used

empty فا

En *empty*
From where data/document is obtainable

empty فا

En *empty*
What processes are involved for a request to access data/document

empty فا

En *empty*
Comments

empty فا

En *empty*

Trial results

Summary result posting date
empty

Table of baseline comparison - English

Table of baseline comparison - Persian

Participant flow diagram - English

Participant flow diagram - Persian

Table of variable outcomes' results - English

Table of variable outcomes' results - Persian

Table of adverse events - English

Table of adverse events - Persian

First publication date
empty

Abstract of published paper

empty فا

En *empty*

Link to full English paper
empty

Link to full Persian paper
empty

- Home (/)
- About IRCT (/)
- Contact us (/)
- Help (/)

Tel:
Working hours:
8:00 - 15:30 Tehran time
11:30 - 19:00 GMT

0098 21 8670 5503

During COVID-19 Epidemic at working times:
0098 936 770 7834

Fax:
0098 21 8670 5503

Email:
admin@irct.ir (mailto:admin@irct.ir)

Directly contacting the manager:
0098 912 778 2686

Address:
IRCT administration team,
Central Library Building, Iran University Campus,
Hemmat freeway, next to Milad tower,
Tehran, 14496-14535
Iran

Copyright © IRCT 2008-2021. All Rights Reserved.
