# Supplementary material for: Comparing the effect of childbirth preparation courses delivered both in-person and via social media on pregnancy experience, fear of childbirth, birth preference and mode of birth in pregnant Iranian women: A quasi-experimental study
Source: PLoS One. 2022 Aug 5;17(8):e0272613. doi: 10.1371/journal.pone.0272613 (PMC9355199; doi:10.1371/journal.pone.0272613)
Supplement: S2 Protocol — (PDF) [file pone.0272613.s003.pdf]

بازگشت (https://fa.irct.ir/user/profile)

مقایسه تأثیر آموزش آمادگی برای زایمان به دو شیوه حضوری و مبتنی بر شبکه اجتماعی بر تجربه بارداری، زایمان و نوع زایمان در زنان باردار

ثبت تایید شده

شناسه کارآزمایی

30890

کد ثبت در IRCT

IRCT20180427039436N2

تاریخ تایید ثبت در مرکز

2018-06-15, ۱۳۹۷/۰۳/۲۵

تاریخ تایید ثبت در مرکز

2018-06-15, ۱۳۹۷/۰۳/۲۵

شماره عضویت

39436

اگر بازخوردی برای داور و نحوه داوری وی، و یا توضیحی در خصوص نحوه ورود اطلاعات خود دارید، لطفا در این قسمت وارد کنید.

ذخیره پیام

اطلاعات عمومی

علت بروز رسانی

فا *خالی*

En *خالی*

عنوان علمی کارآزمایی

فا مقایسه تأثیر آموزش آمادگی برای زایمان به دو شیوه حضوری و مبتنی بر شبکه اجتماعی بر تجربه بارداری، زایمان و نوع زایمان در زنان باردار

En Effect of Childbirth Education based on Face to Face and the Social Network on Pregnancy and Delivery Experience and Type of Delivery on Pregnant Women

عنوان عمومی کارآزمایی

فا بررسی تاثیر آموزش مجازی آمادگی برای زایمان در زنان باردار

En Effect of virtual Childbirth Education on pregnant women

نام اختصاری

خالی

طراحی مطالعه

مداخله‌ای

فاز مطالعه

مصداق ندارد

حداقل سن

year 18

حداکثر سن

year 35

جنسیت

مونث

شرایط عمده ورود به مطالعه قبل از تصادفی سازی

فا داشتن ملیت ایرانی
نخست زا بودن
زنان 18-35 ساله
سن حاملگی ۲۰ هفته
توانایی خواندن و نوشتن
داشتن تلفن همراه یا کامپیوتر با قابلیت نصب برنامه تلگرام تا پایان مطالعه
دسترسی به اینترنت فعال
عدم وجود هر گونه بیماری یا شرایط خاصی که فرد را در گروه بارداری پر خطر قرار دهد
عدم سابقه ناباروری و بیماری های روانی

En Iranian nationality
nullipara
18-35 years old
20 week of gestational age
Literate
Having a mobile phone or computer with the ability to install a telegram program until the end of the study

Active Internet access
Absence of high risk pregnancy
Lack of history of infertility and psychological disorder

شرایط عمده عدم ورود به مطالعه قبل از تصادفی سازی

فا عدم حضور در بیش از ۲ جلسه از کلاس های آمادگی زایمان
ترک گروه به هر دلیلی (انصراف نمونه ها از ادامه شرکت در مطالعه)
بازخورد ندادن نسبت به پیام های ارسال شده توسط پژوهشگر به مدت حداقل یک هفته طی مطالعه
بروز هر گونه علائم خطر بارداری در طی مطالعه که فرد را به گروه پرخطر وارد سازد
زایمان زودرس

En Being absent more than two sessions of childbirth classes
Leave the group
Not responding to messages sent by the researcher for at least one week during the study
Indication of high risk pregnancy during study
Preterm labor

تصادفی سازی (نظر محقق)
اختصاص غیر تصادفی به گروه‌های مداخله و کنترل

توصیف نحوه تصادفی سازی
فا *خالی*
En *خالی*

کور سازی (به نظر محقق)
کور نشده است

توصیف نحوه کور سازی
فا *خالی*
En *خالی*

دارو نما
ندارد

اختصاص به گروه‌های مطالعه
موازی

هدف اصلی مطالعه
آموزشی/مشاوره‌ای

سایر مشخصات طراحی مطالعه
فا *خالی*
En *خالی*

حجم نمونه کل
حجم نمونه پیش‌بینی شده
حجم نمونه پیش‌بینی شده: **165**
.بیش از یک نمونه در هر نفر شرکت کننده: **خیر**
تعداد نمونه در هر نفر شرکت کننده: ***خالی***
توصیف نمونه در هر نفر شرکت کننده - فارسی: فا ***خالی***
توصیف نمونه در هر نفر شرکت کننده - انگلیسی: En ***خالی***

حجم نمونه تحقق یافته
حجم نمونه تحقق یافته: ***خالی***
بیش از یک نمونه در هر نفر شرکت کننده.: **خیر**
تعداد نمونه در هر نفر شرکت کننده: ***خالی***
توصیف نمونه در هر نفر شرکت کننده - فارسی: فا ***خالی***
توصیف نمونه در هر نفر شرکت کننده - انگلیسی: En ***خالی***

تاریخ شروع بیمار گیری مورد انتظار
2018-07-06, ۱۳۹۷/۰۴/۱۵

تاریخ پایان بیمار گیری مورد انتظار
2019-08-21, ۱۳۹۸/۰۵/۳۰

تاریخ شروع بیمارگیری تحقق یافته
خالی

تاریخ پایان بیمارگیری تحقق یافته
خالی

تاریخ خاتمه کارآزمایی
خالی

کد ثبت در سایر مراکز ثبت بین‌المللی

خالی

تاییدیه کمیته‌های اخلاق

کمیته اخلاق

نام کمیته اخلاق
Ethics committee of Iran University of Medical Sciences
کمیته اخلاق دانشگاه علوم پزشکی ایران

آدرس خیابان
Rashid Yasemi
رشید یاسمی

شهر
Tehran
تهران

استان
تهران
کد پستی
1996713883

تاریخ تایید
2018-02-25, ۱۳۹۶/۱۲/۰۶

کد کمیته اخلاق
IR.IUMS.REC 1396.9511373011

بیماری‌های (موضوعات) مورد مطالعه

شرح
فا آموزش مجازی آمادگی برای زایمان
En Virtual education of childbirth preparation

کد ICD-10
خالی

توصیف کد ICD-10
خالی

متغیر پیامد اولیه

شرح متغیر پیامد
فا تجربه بارداری
En pregnancy experience

مقاطع زمانی اندازه‌گیری
فا پیش از مداخله در هفته 20-16 بارداری و در هفته 38-36 بارداری
En Before intervention at 16-20 weeks gestational and 36-38 weeks of gestational age

نحوه اندازه‌گیری متغیر
فا پرسشنامه کوتاه شده تجربه بارداری
En Brief version of pregnancy experience scale

شرح متغیر پیامد
فا انتظار/ تجربه زایمانی

En delivery expectancy/experience questionnaire

مقاطع زمانی اندازه‌گیری

فا پیش از مداخله در هفته 20-16 بارداری و در هفته 38-36 بارداری

En Before intervention at 16-20 weeks gestational and 36-38 weeks of gestational age

نحوه اندازه‌گیری متغیر

فا پرسشنامه انتظار/ تجربه زایمانی

En Wijma delivery expectancy/experience questionnaire

شرح متغیر پیامد

فا نوع زایمان

En Type of delivery

مقاطع زمانی اندازه‌گیری

فا پس از زایمان

En After delivery

نحوه اندازه‌گیری متغیر

فا پرسش مبتنی بر شبکه اجتماعی

En Question based on social networking

متغیر پیامد ثانویه

خالی

گروه‌های مداخله

شرح مداخله

فا آموزش مبتنی بر شبکه اجتماعی (تلگرام): محتوای آموزشی منطبق بر دستورالعمل استاندارد کشوری بوده و ارسال پیام ها بر اساس اصول چندرسانه ای مایر و به صورت متن، تصویر، پادکست و ویدئوکست در فرمت MPEG-4، خواهد بود. حداکثر مدت زمان فیلم های آموزشی، 15-10 دقیقه خواهد بود.

En Education based on social networking (on Telegram): The educational content is according to country standard instructions. In addition, sending messages based on Mayer's multimedia in form of text, image, podcast, video cast, in the MPEG-4 format. The maximum of films time will be 10-15 minute.

طبقه بندی

other

شرح مداخله

فا کلاس های آمادگی زایمان حضوری: این کلاس ها در 8 جلسه به صورت حضوری در بیمارستان برگزار خواهد شد.

En Face to face childbirth preparation classes: These classes will be held at the hospital in 8 sessions.

طبقه بندی

other

شرح مداخله

فا گروه کنترل: عدم شرکت در هر گونه دوره آموزشی

En Control group: Not attending any educational course

طبقه بندی

other

مراکز بیمار گیری

مرکز بیمار گیری

نام مرکز بیمار گیری

Milad hospital

بیمارستان میلاد

نام کامل فرد مسوول

Robab Mousavi

رباب موسوی

آدرس خیابان

Shahid Hemmat highway, District 2

منطقه دو، بزرگراه شهید همت

شهر

Tehran

تهران

استان

تهران

کد پستی

881/14665

تلفن

+98 21 84090

فکس

+98 21 8806 2005

ایمیل

info@miladhospital.com

آدرس صفحه وب

حمایت کنندگان / منابع مالی

حمایت کننده مالی

نام سازمان / نهاد

Iran University of Medical Sciences

دانشگاه علوم پزشکی ایران

نام کامل فرد مسوول

Kazem Malakouti

کاظم ملکوتی

آدرس خیابان

Shahid Hemmat highway, District 2

منطقه دو، بزرگراه همت

شهر

Tehran

تهران

استان

تهران

کد پستی

1449614535

تلفن

+98 21 6650 9024

فکس

+98 21 8805 2248

ایمیل

malakoutik@yahoo.com

ردیف بودجه

فا *خالی*

En *خالی*

کد بودجه

خالی

عنوان منبع مالی

فا دانشگاه علوم پزشکی ایران

En Iran University of Medical Sciences

درصد تامین مالی مطالعه توسط این منبع
100%

بخش عمومی یا خصوصی
public

مبدا اعتبار از داخل یا خارج کشور
domestic

فرد مسوول پاسخگویی عمومی کارآزمایی

نام سازمان / نهاد
Iran University of Medical Sciences

دانشگاه علوم پزشکی ایران

نام کامل فرد مسوول
Robab Mousavi

رباب موسوی

موقعیت شغلی
Student

دانشجو

آخرین مدرک تحصیلی
فوق لیسانس

سایر حوزه‌های کاری/تخصص‌ها
Midwifery

مامایی

آدرس خیابان
Rashid Yasemi

رشید یاسمی

شهر
Tehran

تهران

استان
تهران

کد پستی
1996713883

تلفن
+98 21 4365 1000

تلفن همراه

+98 910 924 5921
ایمیل
rrr.mousavi@gmail.com

فرد مسوول پاسخگویی علمی مطالعه

نام سازمان / نهاد
Iran University of Medical Sciences
دانشگاه علوم پزشکی ایران
نام کامل فرد مسوول
Robab Mousavi
رباب موسوی
موقعیت شغلی
Student
دانشجو
آخرین مدرک تحصیلی
فوق لیسانس
سایر حوزه‌های کاری/تخصص‌ها
Midwifery
مامایی
آدرس خیابان
Rashid Yasemi
رشید یاسمی
شهر
Tehran
تهران
استان
تهران
کد پستی
1996713883
تلفن
+98 21 4365 1000
تلفن همراه
+98 910 924 5921
ایمیل
rrr.mousavi@gmail.com

فرد مسوول به‌روز رسانی اطلاعات

نام سازمان / نهاد
Iran University of Medical Sciences
دانشگاه علوم پزشکی ایران
نام کامل فرد مسوول
Leila Amiri Farahani
لیلا امیری فراهانی
موقعیت شغلی
Assistant professor
استادیار
آخرین مدرک تحصیلی
Ph.D.
سایر حوزه‌های کاری/تخصص‌ها
Reproductive Health
سلامت باروری
آدرس خیابان
Rashid Yasemi
رشید یاسمی
شهر
Tehran
تهران
استان
تهران
کد پستی
1996713883
تلفن
+98 21 4365 1139
تلفن همراه
+98 912 450 5862
ایمیل
l.amirifarahani@gmail.com

چکیده پروتکل

هدف ازمطالعه

فا مقایسه تأثیر آموزش آمادگی برای زایمان به دو شیوه حضوری و مبتنی بر شبکه اجتماعی بر تجربه بارداری، زایمان و نوع زایمان با گروه کنترل در زنان باردار

En Comparing the Effect of Childbirth Education based on Face to Face and the Social Network on Pregnancy and Delivery Experience and Type of Delivery on Pregnant Women

شرکت کنندگان/شرایط ورود و عدم ورود

فا زنان نخست زای واجد شرایط دریافت کننده مراقبت های معمول بارداری

En Nulliparous women are eligible to receive common perinatal cares

گروه‌های مداخله

فا در این مطالعه نمونه ها به طور مساوی، در سه گروه مداخله یک (آموزش مبتنی بر شبکه اجتماعی (تلگرام))، مداخله دو (کلاس های آمادگی زایمان حضوری) و کنترل (عدم شرکت در هر گونه دوره آموزشی) قرار خواهند گرفت.

En In this study, participants will assign to one of three groups: intervention group 1 (education based on social networking (on Telegram)), intervention group 2 (face to face childbirth preparation classes) and control group (Not attending any educational course.

طراحی

فا مطالعه نیمه تجربی دارای گروه کنترل، با سه گروه های موازی، حجم نمونه 165 نفر

En Semi experimental study with control group, with three parallel groups design of 165 patients

نحوه و محل انجام مطالعه

فا این مطالعه در بخش پری ناتال بیمارستان میلاد انجام خواهد شد. محتوای آموزشی مطالب به اشتراک گذاشته شده منطبق با دستورالعمل استاندارد کشوری بوده و ارسال پیام ها بر اساس اصول چندرسانه ای مایر و به صورت متن، تصویر، پادکست و ویدکست در فرمت MPEG-4، خواهد بود. حداکثر مدت زمان فیلم های آموزشی، ۱۵-۱۰ دقیقه بوده و جهت جلوگیری از ارسال یکباره مطالب آموزشی، محتوای هر جلسه در بخش های تقسیم بندی شده، به صورت روزانه ارسال خواهد شد. جهت اطمینان از آموزش صحیح تکنیک های تنفسی و تن آرامی، دو جلسه برای تمرین حضوری تشکیل خواهد شد.

En This study will be conducted in the perinatal clinic of the Milad Hospital. The educational content is according to country standard instructions. In addition, sending messages based on Mayer's multimedia in form of text, image, podcast and video cast, in the MPEG-4 format. The maximum of films time was 10-15 minute. To prevent from being uploaded, as well as reduction in educational quality, the content of each session in divided sections in that session and specific time will be sent every day. To make sure of correct methods education of respiratory and relaxation techniques, two sessions to practice will be hell in the hospital.

متغیرهای پیامد اصلی

فا تجربه بارداری؛ انتظار/تجربه زایمانی؛ تمایل به نوع زایمان؛ نوع زایمان

En Pregnancy experience; Delivery expectancy/experience; Desire to type of delivery; Type of delivery

برنامه انتشار

فایل داده شرکت کنندگان (IPD)
هنوز تصمیم نگرفته‌ام - برنامه انتشار آن هنوز مشخص نیست
توجیه/علت عدم تصمیم/عدم انتشار IPD
فا در اینده تصمیم گیری می شود.
En it is will be known in future.

پروتکل مطالعه
خیر - برنامه‌ای برای انتشار آن وجود ندارد
نقشه آنالیز آماری
خیر - برنامه‌ای برای انتشار آن وجود ندارد
فرم رضایتنامه آگاهانه
خیر - برنامه‌ای برای انتشار آن وجود ندارد
گزارش مطالعه بالینی
خیر - برنامه‌ای برای انتشار آن وجود ندارد
کدهای استفاده شده در آنالیز
خیر - برنامه‌ای برای انتشار آن وجود ندارد
نظام دسته‌بندی داده (دیکشنری داده)
خیر - برنامه‌ای برای انتشار آن وجود ندارد
عنوان و جزییات بیشتر در مورد داده/مستند
فا *خالی*
En *خالی*

بازه زمانی امکان دسترسی به داده/مستند
فا *خالی*
En *خالی*

کسانی که اجازه دارند به داده/مستند دسترسی پیدا کنند
فا *خالی*
En *خالی*

به چه منظور و تحت چه شرایطی داده/مستند قابل استفاده است
فا *خالی*
En *خالی*

برای دریافت داده/مستند به چه کسی یا کجا مراجعه شود
فا *خالی*
En *خالی*

یک درخواست برای داده/مستند چه فرایندی را طی می‌کند
فا *خالی*
En *خالی*

سایر توضیحات
فا *خالی*
En *خالی*

نتایج مطالعه کارآزمایی

تاریخ اضافه کردن خلاصه نتایج
خالی

جدول مقایسه اطلاعات پایه در گروه‌ها - انگلیسی

جدول مقایسه اطلاعات پایه در گروه‌ها - فارسی

دیاگرام مراحل وارد شدن شرکت کنندگان به مطالعه - انگلیسی

دیاگرام مراحل وارد شدن شرکت کنندگان به مطالعه - فارسی

جدول نتایج متغیرهای پیامد - انگلیسی

جدول نتایج متغیرهای پیامد - فارسی

جدول رخدادهای نامطلوب - انگلیسی

جدول رخدادهای نامطلوب - فارسی

تاریخ چاپ اولین مقاله

خالی

چکیده مقاله منتشر شده

فا *خالی*

En *خالی*

پیوند به لینک انگلیسی

خالی

پیوند به لینک فارسی

خالی

- خانه (/)
- درباره IRCT (/)
- تماس با ما (/)
- راهنما (/)

تلفن‌های مرکز:

ساعت تماس: ۸:۰۰ الی ۱۵:۳۰

۰۰۹۸-۲۱-۸۶۷۰-۵۵۰۳

در طی اپیدمی کرونا در ساعات اداری روزهای کاری:
۰۰۹۸-۹۳۶-۷۷۰-۷۸۳۴

فکس:
۰۰۹۸-۲۱-۸۶۷۰-۵۵۰۳

ایمیل:
admin@irct.ir (mailto:admin@irct.ir)

تماس مستقیم با تلفن شخصی مدیر:
۰۰۹۸-۹۱۲-۷۷۸-۲۶۸۶

آدرس:
مرکز ثبت کارآزمايي هاي باليني ايران،
پردیس دانشگاه علوم پزشکی ایران،
ساختمان کتابخانه مرکزی،
بزرگراه همت، جنب برج میلاد،
کد پستی: ۱۴۵۳۵-۱۴۴۹۶
تهران، ایران.

کلیه حقوق این سایت به مرکز ثبت کارآزمایی بالینی ایران تعلق دارد.
