# Supplementary material for: Comparing the effect of childbirth preparation courses delivered both in-person and via social media on pregnancy experience, fear of childbirth, birth preference and mode of birth in pregnant Iranian women: A quasi-experimental study
Source: PLoS One. 2022 Aug 5;17(8):e0272613. doi: 10.1371/journal.pone.0272613 (PMC9355199; doi:10.1371/journal.pone.0272613)
Supplement: Attachment — Submitted filename: PlosONE REVIEW FOC 22.2.22.docx [file pone.0272613.s004.docx]

PlosONE REVIEW	Carmen Power 22.2.22

Short title: Social media-based training and pregnancy and childbirth outcome

ABSTRACT – note the whole text here is repeated twice

Results section: Give percentages of vaginal birth and elective CS for different study groups otherwise the conclusion doesn’t make sense as it’s not a summary of the results section.

*Ethical permission and written consent – yes

SUMMARY

Expand limitations section – Was the drop-out rate different between groups? If so, this in itself could be a suggestion that women prefer to attend in-person/online pregnancy/birth prep classes.

INTRODUCTION

Check meaning in sentence 2 – repeat of childbirth preparation courses after ‘provide’.

METHOD

Good use of validated questionnaires. Description of statistical methods is good.

*A few things to think about and possibly discuss*:

1. I’m not sure I understand why the women were ‘non-randomly’ allocated to the 3 groups? How did this prevent their awareness of the other groups if they were choosing their group? And think about how might this have affected the results as it could mean that women who wanted a vaginal birth were more likely to choose a certain group and vice versa. Having studied the flow chart, perhaps you meant to say they were randomly allocated to groups? (i.e. neither the participant nor researcher could show any bias)
2. Could the content involved in online prep (5-15 mins) sessions be linked to it being available on a daily basis so women could watch videos repeatedly, remembering and reinforcing the information?
3. Did Group A having a 2 x 2 hour face to face component blur the findings at all?
4. Did Group B also receiving videos and music blur the difference between the 2 groups?

RESULTS

1. Think about showing your full statistical workings and outcomes in the tables.
2. Clarify in Table 2 (and other tables where needed) whether bracketed numbers are the standard deviation or a percentage of the non-bracketed number directly before.
3. P19 – text underneath table 4 is confusing – please clarify your meaning here – what did the statistical outcomes signify?

DISCUSSION

1. The findings are important in that FOC is a crippling psychological condition that can results in unnecessary interventions. Could a larger sample size be used in a future study?
2. It might also be mentioned that a mixture of online and face to face classes is more manageable for pregnant women who have other children or who have to travel.

GENERAL

Ideally this paper needs to be proofread and edited by a fluent English speaker as there are differences in grammar and turns of phrase that interfere with a smooth reading process. More importantly, the semantics are sometimes lost and your results could be misinterpreted during reading although ultimately they become clear.
